# Transmission of *Leishmania donovani* in the Hills of Eastern Nepal, an Outbreak Investigation in Okhaldhunga and Bhojpur Districts

**DOI:** 10.1371/journal.pntd.0003966

**Published:** 2015-08-07

**Authors:** Bart Ostyn, Surendra Uranw, Narayan Raj Bhattarai, Murari L. Das, Keshav Rai, Katrien Tersago, Yubraj Pokhrel, Lies Durnez, Baburam Marasini, Gert Van der Auwera, Jean-Claude Dujardin, Marc Coosemans, Daniel Argaw, Marleen Boelaert, Suman Rijal

**Affiliations:** 1 Department of Public Heath, Institute of Tropical Medicine, Antwerp, Belgium; 2 Department of Internal Medicine, B.P. Koirala Institute of Health Sciences, Dharan, Nepal; 3 Department of Microbiology, B.P. Koirala Institute of Health Sciences, Dharan, Nepal; 4 Epidemiology and Disease Control Division, Ministry of Health and Population (MOHP), Kathmandu, Nepal; 5 Department of Biomedical Sciences, Institute of Tropical Medicine, Antwerp, Belgium; 6 Department of Biomedical Sciences, University of Antwerp, Antwerp, Belgium; 7 Department of Control of Neglected Tropical Diseases, World Health Organization (WHO), Geneva, Switzerland; The Hebrew University of Jerusalem, ISRAEL

## Abstract

**Background:**

In the Indian subcontinent, Visceral leishmaniasis is endemic in a geographical area coinciding with the Lower Gangetic Plain, at low altitude. VL occurring in residents of hill districts is therefore often considered the result of *Leishmania donovani* infection during travel. Early 2014 we conducted an outbreak investigation in Okhaldhunga and Bhojpur districts in the Nepal hills where increasing number of VL cases have been reported.

**Methodology/Principal Findings:**

A house-to-house survey in six villages documented retrospectively 35 cases of Visceral Leishmaniasis (VL). Anti-Leishmania antibodies were found in 22/23 past-VL cases, in 40/416 (9.6%) persons without VL and in 12/155 (7.7%) domestic animals. An age- and sex- matched case-control study showed that exposure to known VL-endemic regions was no risk factor for VL, but having a VL case in the neighbourhood was. SSU-rDNA PCR for Leishmania sp. was positive in 24 (5%) of the human, in 18 (12%) of the animal samples and in 16 (14%) bloodfed female *Phlebotomus argentipes* sand flies. *L*. *donovani* was confirmed in two asymptomatic individuals and in one sand fly through hsp70-based sequencing.

**Conclusions/Significance:**

This is epidemiological and entomological evidence for ongoing local transmission of *L*. *donovani* in villages at an altitude above 600 meters in Nepal, in districts considered hitherto non-endemic for VL. The VL Elimination Initiative in Nepal should therefore consider extending its surveillance and control activities in order to assure VL elimination, and the risk map for VL should be redesigned.

## Author Summary

Visceral leishmaniasis is a neglected but deadly disease occurring in north-eastern India, the south-eastern lowland of Nepal, and the Ganges delta in Bangladesh; all part of the Lower Gangetic plains. Districts at higher altitude, such as those situated in the foothills of the Himalaya in Nepal, are considered non-endemic. Consequently, diagnostic, therapeutic and surveillance facilities are not available, and sporadic cases of VL occurring in residents of these districts are considered the result of *Leishmania donovani* infection during travel. This parasite is transmitted from man to man through the bite of a sand fly, *Phlebotomus argentipes*. To investigate the increasing number of cases reported from some villages in the Nepal hills, we interviewed former VL cases, as well as their healthy co-villagers, on their history of travel, we checked their blood for signs of infection, and we set traps to capture sand flies. We found that many residents had been infected, symptomatically as well as asymptomatically, regardless of travelling to endemic areas. Moreover, we managed to capture sand flies and found DNA of leishmania parasites inside. This proves that there is indeed ongoing local transmission in hill districts and that surveillance and control activities should be extended.

## Introduction

Visceral Leishmaniasis (VL) is a vector-borne parasitic disease that is fatal if left untreated. With an estimated 162,000 to 313,000 new cases per year in northeast India, Nepal and Bangladesh, VL poses a public health problem in the Indian subcontinent (ISC) [1]. In this region, VL is caused by *Leishmania donovani* and transmitted by *Phlebotomus argentipes* with humans as the only reservoir [2]. In endemic foci, infected domestic animals have been encountered, clustering with asymptomatic human infections, but their role in transmission is not established [3]. The habitat of the sand fly vector depends on biotic (vegetation and availability of human and/or animal blood meals) and abiotic (temperature and precipitation) factors, specific for each species. In the case of *P*. *argentipes*, these conditions are met in the plains of the river Ganges. The range of this species is considered to be limited to altitudes below 700 meters above the sea level [4–6].

In Nepal, the disease is endemic in 12 districts in the south-eastern plains, known as the *Terai*, where eight million people are at risk. Sporadic VL cases were reported in the hills at altitudes of 1,000 meters above sea level [7–8], but none of these have been firmly linked to local transmission. As the travel history of these cases was not documented, they may possibly have contracted VL in the Terai plains. Transmission of VL in the foothills of the Himalaya at altitudes ranging from 1,300 to 3,000 m has been suggested elsewhere, in India [9–12] and in Bhutan [13]—though not unequivocally confirmed as local. The transmission cycle requires a large vector population, but only few of these studies include entomological information [13–14]. Ozbel *et al*. suggested that not the altitude itself, but the gradient of habitats, relief and climate it offers, exerts the structuring effect on sand fly range [15].

Since 2000, VL cases have been reported in increasing numbers from the hilly regions in eastern Nepal. This challenges the ongoing VL elimination program—as they could constitute a reservoir of future re-emergence if local transmission is confirmed. We report an outbreak investigation conducted early 2014 in this region.

## Methods

### Selection of study districts

We reviewed the epidemiological surveillance data of the Ministry of Health & Population and the patient database of the BPKIHS hospital for the period 2000–2013. All VL cases from the last five years who named a hill district as their residence at the time of admission were listed and the two most affected districts, namely Bhojpur and Okhaldhunga, were selected for field investigation.

### Selection of study clusters

In each district we selected three villages on the basis of the total number of VL cases since 2005, combined with the number of cases reported during each of the previous two years, accessibility on foot and local support by health authorities and community. The selected locations were Thakle Jakme, Thakle Richuwa and Mathilo-Richuwa in Okhaldhunga district, and Bhojpur village ward no.3, Dalgaun ward no.3, and Manebhanjyang ward no.9 from Bhojpur district.

### Case ascertainment

The following case definitions were used: **current VL case**: fever for at least two weeks and positivity in a rapid diagnostic test for VL (Kala-azar Detect TM Rapid Test; InBios International, Seattle, WA); **past case of VL**: history of treatment for VL, corroborated by prescriptions and/or case records from the health facility; **past VL death**: any death related to a febrile illness of longer than two weeks duration and at least one more VL-specific sign as verified by a verbal autopsy procedure.

### Field study

The investigation took place in March 2014 in Okhaldhunga district, and in May 2014 in Bhojpur. We conducted an exhaustive household census combined with a serosurvey, a case-control study, and an entomological survey.

**Household census.** All households were geo-referenced by a Global Positioning System (GPS) device, specifying longitude, latitude and altitude of the houses. Trained field workers interviewed each head of household on age and gender of present and absent family members, occupation, household characteristics, and livestock ownership, as well as current or past history of VL in the family including deaths. For each VL case, disease history, health care-seeking behavior, place of treatment, drugs and outcome were recorded.

**Case-control study.** We interviewed each current or past VL case identified in the census with a semi-structured questionnaire to assess risk factors. For each case we selected four age- and sex-matched controls (age matching based on age at time of VL diagnosis) living in the same village without history of VL in their lifetime, and applied the same questionnaire. The power of this nested case-control study was calculated prior to the study with the factor “contact with known VL endemic area” as primary exposure of interest, and VL as the outcome. Based on the expectation to find at least 35 cases, a ratio of four controls per case allowed to detect an odds ratio of three with a power of 80% at a significance level of 5%.

The risk factor questionnaire focused on both individual and household characteristics such as educational status, occupation, travel history, sleeping habits, bed net use, cases and deaths due to VL in the family and in the immediate neighborhood, socio-economic factors, animal ownership and housing characteristics.

**Serological study.** All subjects aged ≥ 2 years who lived permanently in the study clusters—or their guardians—were invited to give consent for the draw of a 2 ml venous blood sample. A trained laboratory technician collected a 2 ml venous blood sample that was divided over an EDTA tube, a serum tube and onto a pre-printed Whatman # 3 filter paper. The dried filter papers were placed in plastic bags containing silica gel. All samples were transported in a cold box at 4–8°C until storage at -20°C at BPKIHS. At the same time, veterinary technicians took blood samples from domestic animals such as bovines, goats, sheep, pigs, and dogs.

**Entomology.** Timing of the insect collections (March-May) purposely coincided with the second annual peak of *P*. *argentipes* density in the plains [16]. Sand flies were captured in-door in eight households including houses of past VL patients, in each of the six study clusters. Trained insect collectors supervised by an experienced medical entomologist installed CDC light traps before dusk inside the house and/or cattle shed for two consecutive nights and collected them between 5:00 and 6:00 AM the next morning. During that visit they also caught resting sand flies by mouth aspiration for 15 minutes in the households and the cattle sheds. Collected specimens were preserved the same day in 80% ethanol and transported to the entomology laboratory at BPKIHS Dharan for examination under a binocular dissecting microscope. Insect species were morphologically identified according to the Lewis key [17] and female *P*. *argentipes* were separated from other insects and pooled by household in a cryotube with 80% alcohol. During further processing for molecular analyses, the source material was completely blinded with regard to species, sex and feeding/gravid status.

**Direct agglutination test (DAT).** DAT was performed using a freeze-dried antigen of fixed, trypsin-treated and stained promastigotes of *Leishmania donovani* obtained from ITM-Antwerp as described by Jacquet *et al*. [18]. A DAT titre ≥ 1:1600 in capillary blood was taken as marker of *Leishmania donovani* infection in humans [19].

**PCR-based detection of *Leishmania sp* and species identification.** DNA was extracted from all blood samples and sand flies, using the QiaAmp DNA mini kit (Qiagen, Hilden, Germany). DNA from 200 μl blood or single sand flies was eluted in 50 μl AE buffer. A diagnostic *Leishmania* PCR based on the small-subunit ribosomal DNA of *Leishmania*, (SSU-rDNA) was performed on 2 μl DNA of each sample, with the inclusion of an internal control amplicon, essentially as described in Odiwuor et al. [20]. A sample was considered to contain *Leishmania* sp. if the PCR scored positive, i.e. if an amplicon of around 115 bp was seen on an ethidium-bromide stained agarose gel. Other samples were scored negative when only the internal control amplicon was successfully amplified, or invalid in case the internal control amplicon was not detected even after repeating the PCR. To verify the *Leishmania* sp. identity of the SSU-rDNA amplicons, they were sequenced and compared with publically available sequences. In order to further type the parasites to the *L*. *donovani* species level, the heat-shock protein 70 gene (*hsp70*) was partially amplified, using HSP70-F as outer and HSP70-N as hemi-nested PCR [21], followed by sequence analysis [22–23].

All sand flies that were positive for the SSU-rDNA PCR were molecularly identified by sequencing the barcoding fragment of the mitochondrial cytochrome oxidase I (COI) as described by Versteirt *et al*. [24]. Sequences were submitted to the online identification tool of the Barcoding of Life Database (BOLD) to identify the sand fly [25].

### Data analysis

We used R statistical software version 3.0.0. [26] for data analysis. For each potential risk factor we computed Odds Ratios by means of a logistic regression adjustment analysis with matching variables (age, gender, village) forced into the regression model.

To retain only the independent predictors of being a VL case, statistically significant risk factors in the univariate analyses (P < 0.05) were entered in a multivariate analysis. The final model and adjusted Odds Ratio’s resulted from a stepwise selection in a logistic regression analysis with matching (age, gender, village) adjustment [27].

### Ethical considerations

Ethical clearance for the study was obtained from the Institutional Ethical Review Boards of BPKIHS and the Nepal Health Research Council, Kathmandu in Nepal, the Ethical Review Board of the Institute of Tropical Diseases in Antwerp, and the University Hospital of Antwerp in Belgium. Community consent was sought and obtained during meetings with local health facilities’ staff, village authorities, and village assemblies. Individual informed consent was obtained in writing, prior to blood sampling and interviewing. For children, a parent or guardian provided written informed consent.

## Results

### VL cases and risk factors

**Fig 1:**
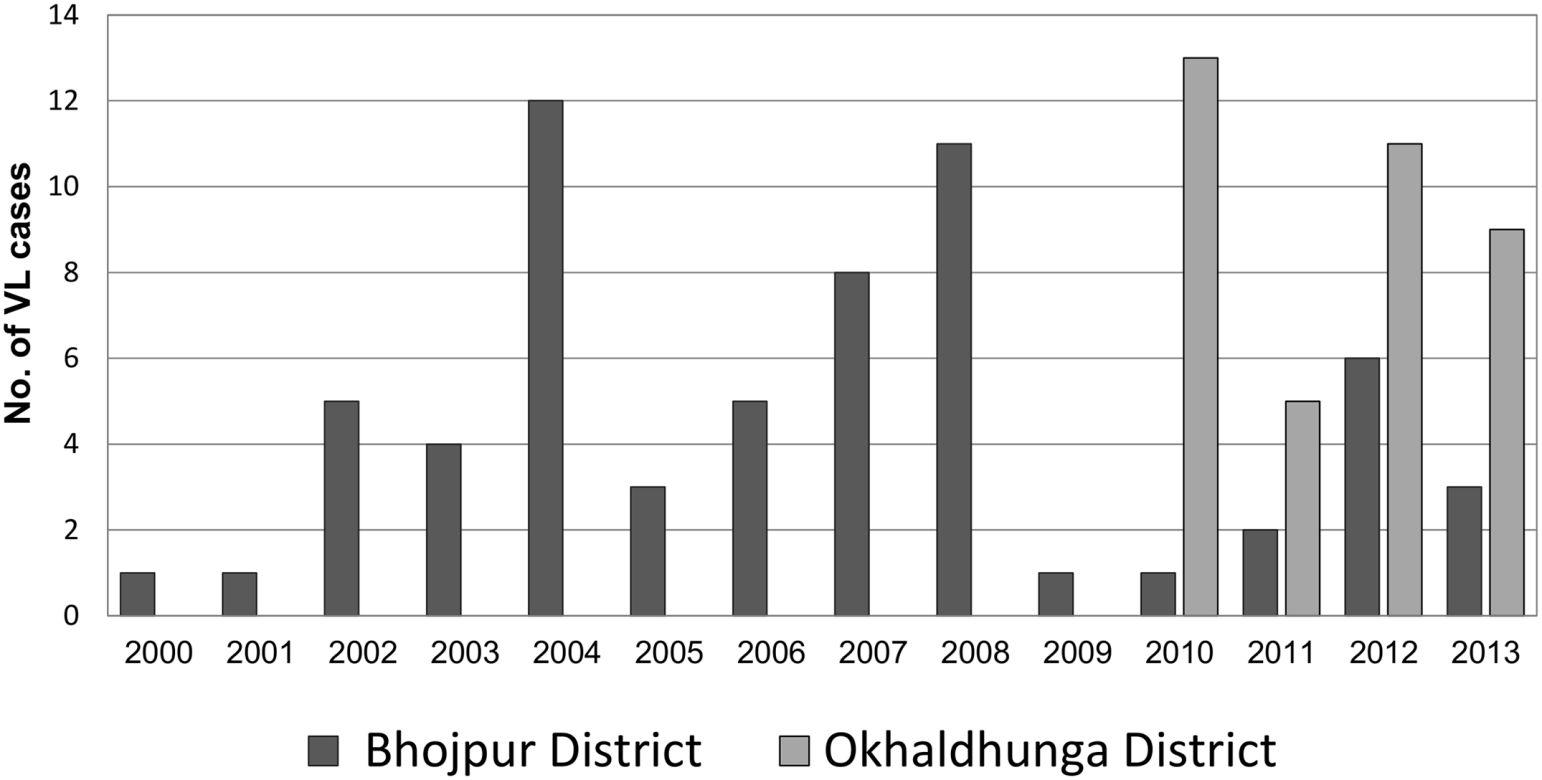
Reported VL case load 2000–2013, Bhojpur and Okhaldhunga Districts, Nepal. n = 101 Sources: Patient database of BPKIHS Dharan hospital & EDCD Teku, Kathmandu.

We retrieved 101 past VL cases in the document review, 38 from Okhaldhunga and 63 from Bhojpur district. The distribution over time of these cases is shown in Fig 1. In Okhaldhunga cases were only reported after 2010, when the mission hospital there started outreach activities, including screening for VL.

The characteristics of the six study clusters are shown in Table 1. The characteristics of the households are included as supporting information (S1 Fig and S1, S2 and S3 Tables).

**Table 1:** Characteristics of study clusters.

District	Study clusters	HHs^a^	Altitude (m)	Population size^b^	Population present (%)	Blood samples collected	Past VL
Okhaldhunga	Thakle Jakma	26	536–652	160	115 (72%)	111	3
	Thakle Richuwa	37	378–717	199	136 (68%)	129	12
	Mathilo Richuwa	34	1026–1213	231	122 (53%)	114	10
Bhojpur	Dalgaun-3	7	1052–1110	33	25 (76%)	25	1
	Manebhanjyang-9	5	449–459	28	22 (79%)	22	7
	Bhojpur-3	13	1532–1583	56	44 (79%)	42	2
**TOTAL**		**122**		**707**	**464 (66%)**	**443**	**35**

^a^HH: household.

^b^Includes family members who were temporarily absent at the time of the census.

No active VL cases were found in the household survey, but 21 percent (26/122) of the households had at least one confirmed case of VL in the past. We ascertained a total of 35 confirmed VL cases, of which four had occurred before the year 2000, and one of them more than 50 years ago. Age at time of VL ranged from 1 to 52 years, most frequent in the youngest age group (Q1/Median/Q3 was 6, 17 and 28 years resp.) (see S2 Fig in supplementary material). Of the 31 VL cases reported since 2000, 22 (71%) were reported in the last five years. Fourteen of these 31 had their diagnostic workup at the BPKIHS-hospital in Dharan where they were parasitologically confirmed as VL. In one of them, who was enrolled in a research project, *L*. *donovani* was confirmed at species level through *hsp70* sequencing of an isolate cultured from a bone marrow aspirate.

Of the 35 confirmed VL cases, four had been fatal, three of which were children below five years old. Treatment comprised Sodium-Antimony-Gluconate (13), miltefosine (5) or amphotericin B (17). An additional four deaths were recorded that could possibly be attributed to VL according to the verbal autopsy, and two of them had a confirmed VL case among other family members. These four persons were not included as VL cases in further analysis.

Table 2 shows the breakdown by year of onset of all the VL cases retrieved during the house-to-house survey.

**Table 2:** Number of VL (KA) cases retrieved in the study clusters over study period.

Study clusters	Population^a^	1964–2000	2001–2004	2005–2009	2010–2013	Total no. VL cases
Thakle Jakma	160	0	0	0	3	3
Thakle Richuwa	199	3	0	3	6	12
Mathilo Richuwa	231	0	0	4	6	10
Dalgaun WN # 3	33	0	0	1	0	1
Manebhanjyang- 9	28	0	3	2	2	7
Bhojpur WN # 3	56	1	1	0	0	2
**TOTAL**	**707**	**4**	**4**	**10**	**17**	**35**

^a^Currently present in the village.

Sex-, age- and village-adjusted Odds Ratios of potential risk factors for being a VL case are given in Table 3. History of travelling to any of the known VL endemic areas was not associated with being a VL case (Odds Ratio 0.73 [95% CI 0.27–1.87]. The strongest risk factor for being a VL case was the occurrence of a VL case in the neighborhood (i.e. within a perimeter of 100 meter from the house). All the 35 (100%) of the VL cases reported such a case in the neighborhood against 52/139 (37 percent) of the controls, leading to an Odds Ratio of positive infinity. Other significant risk factors for VL in univariate analysis were cow dung in front of the house, sleeping on the ground, increasing distance from the river, monthly income, house type and having a case of VL in the family. A multivariate stepwise logistic regression modeling procedure—ignoring the factor “VL case in neighborhood”—retained only sleeping on the ground, (OR 5.65 [95% CI 1.12–30.43], cow dung in front of the house (OR 4.70 [95% CI 1.21–20.06]) and distance from the river (OR 0.06 per additional km from river [95% CI 0.001–0.92]) as independent predictors. As these ORs could be confounded by the effect of having a VL case in the neighborhood, a factor that could not be included in the multivariate modeling approach because of its infinite OR, we did a stratified analysis on a restricted data set that only included those cases and controls with a VL case in the neighborhood. Beyond that factor only two lifestyle-related risk factors remain statistically significant: cow dung in front of the house (OR 4.72 [95% CI 1.64–15.26] and sleeping on the ground (OR 4.74 [95% CI 1.18–21.5]).

**Table 3:** Number of cases and controls with potential risk factors for KA, univariate Odds Ratios (OR) controlled for age, sex, village and adjusted OR obtained in final multivariate model.

	Cases	Controls	Univariate			Multivariate		
	(n = 35)	(n = 139)	OR^a^	95% C.I.	*p*	OR^b^	95%CI	*p*
Contact with known endemic areas	9	40	0.73	0.27–1.87	*0*.*524*			
VL case in family	15	26	3.21	1.26–8.23	*0*.014*			
VL case in close^d^ neighborhood	35	52	116^e^	6.90–1915.42	*0*.000***			
DAT-positive in family	20^c^	47	2.36	0.96–5.89	*0*.*061*			
Monthly income (per 1000 NRS)			1.11	1.09–1.22	*0*.035*			
Occupation					*0*.*421*			
Agriculture (ref)	4	19	1	-	-			
Daily labour	7	12	2.72	0.58–14.27	*0*.*211*			
House wife	8	24	2.09	0.46–11.34	*0*.*357*			
Student	14	77	0.81	0.16–4.99	*0*.*809*			
Other	2	7	-	-	-			
House type					*0*.*096*			
Thatched (ref)	11	52	1	-	-			
Mud (Kaccha)	17	61	2.14	0.63–8.73	*0*.*246*			
Semi-Pakka (Mixed cement)	6	14	5.59	1.17–29.31	*0*.033*			
Wood	1	12	-	-	-			
Sleeping on the ground	16	34	3.60	1.23–10.72	*0*.020*	5.65	1.12–30.43	*0*.*035*
Sleeping under bed net	6	33	0.60	0.19–1.71	*0*.*369*			
Owning domestic animal(s)	29	104	0.60	0.55–1.09	*0*.*274*			
Cow dung in front of house	21	42	4.01	1.72–9.65	*0*.001**	4.70	1.21–20.06	*< 0*.*001*
Vegetation around house	11	39	0.86	0.19–3.21	*0*.*837*			
Contact with river	14	27	2.92	0.85–10.25	*0*.*088*			
Distance house- river (per km)			0.06	0.002–0.53	*0*.010*	0.06	0.001–0.92	*0*.*001*

^a^Adjusted for age, sex, village in separate logistic regression models.

^b^Adjusted OR obtained in final multivariate conditional logistic regression model.

^c^n = 34.

^d^Within 100 meter from house.

^e^OR = positive infinity. Values shown were computed for 34,9999 exposed cases instead of 35.

### *L. donovani* infection

**Fig 2:**
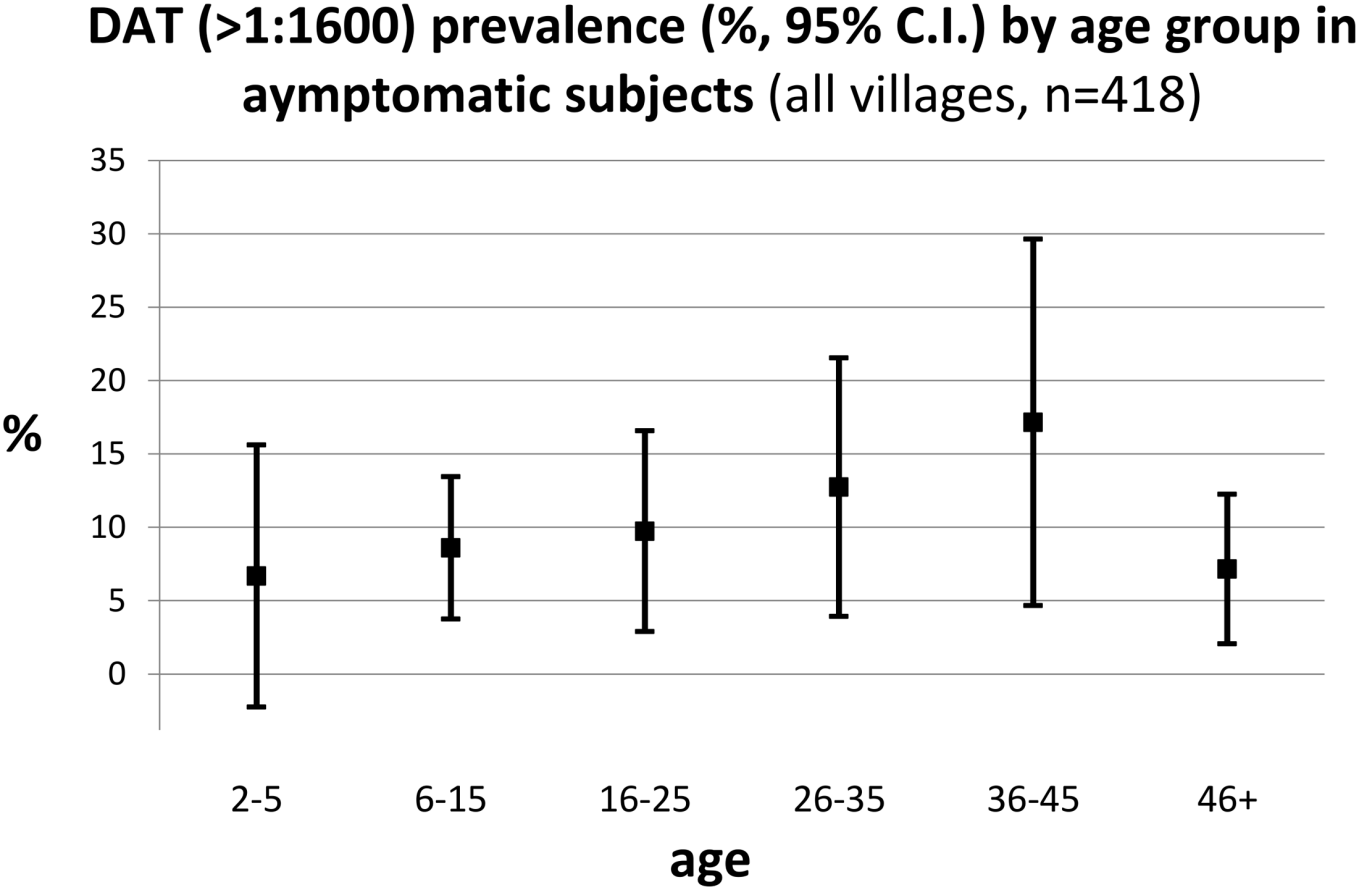
Seroprevalence by age group.

Overall, 14.1% (95% CI: 10.8–17.3) of the study population (62/441) was positive in DAT varying from 2.5% to 50% according to study cluster. Excluding the past VL cases, seroprevalence in the asymptomatic persons was 9.6% (95% CI: 6.8–12.4) varying between villages from 0 to 42%. There was no significant difference in seroprevalence according to sex and age (Fig 2).

Blood samples were collected from 155 domestic animals. Ten goats, one cow and one buffalo had DAT titers just above the cut-off (one (goat) = 1/6400, all others = 1/1600). Of all goats sampled (n = 83), this represented 12%. DNA was extracted from human (n = 441) and animal blood samples (n = 155). SSU-rDNA was positive in 24 (5%) of the human samples, and in 18 (12%) of the animal samples. The amplicon sequence was obtained from five humans, four goats, and one sheep, and in all cases, the sequence was compatible with *Leishmania sp*. An *hsp70* sequence could be obtained from two of the human samples, which confirmed the parasite identity as *Leishmania donovani*. The *hsp70* gene from the animal samples could not be sequenced, likely because of the low parasitemia in these samples, together with the lower sensitivity of the hsp70 PCR (vs the SSU-rDNA PCR).

### Entomology

The total number of sand flies captured was 281, morphologically identified and segregated into *P*. *argentipes*, *Sergentomyia spp*. and other *Phlebotomus spp*. Amongst the *P*. *argentipes*, 82 were male, 29 unfed female, 108 fed female, and 6 gravid female. DNA extraction was successful in 271 sand flies and 16 specimens (14,5% of the female, fed sand flies) were found positive by SSU-rDNA PCR. In 11 of these the sequence could be obtained and was found compatible with *Leishmania sp*. The *hsp70* sequence was obtained from one of these, confirming infection with *L*. *donovani*. 14 out of 16 SSU-rDNA PCR positive sand flies, including the one infected with *L*. *donovani*, were molecularly identified as *P*. *argentipes*, and all of them were female and fed. The other two were confirmed to be *Phlebotomus* spp., but could not be molecularly identified to species level as their *COI* sequence was not available in BOLD. Retracing the origin of the 16 sand flies, they came from 10 houses, i.e. two households from Thakle Jakma, five from Thakle Richuwa, two from Mathilo (Okhaldhunga district), and one from a household in Manebhanjyang (Bhojpur). Five of these households effectively had had a VL case in the past, one had asymptomatic DAT positives only, and four had neither (though not all family members gave a blood sample).

**Fig 3:**
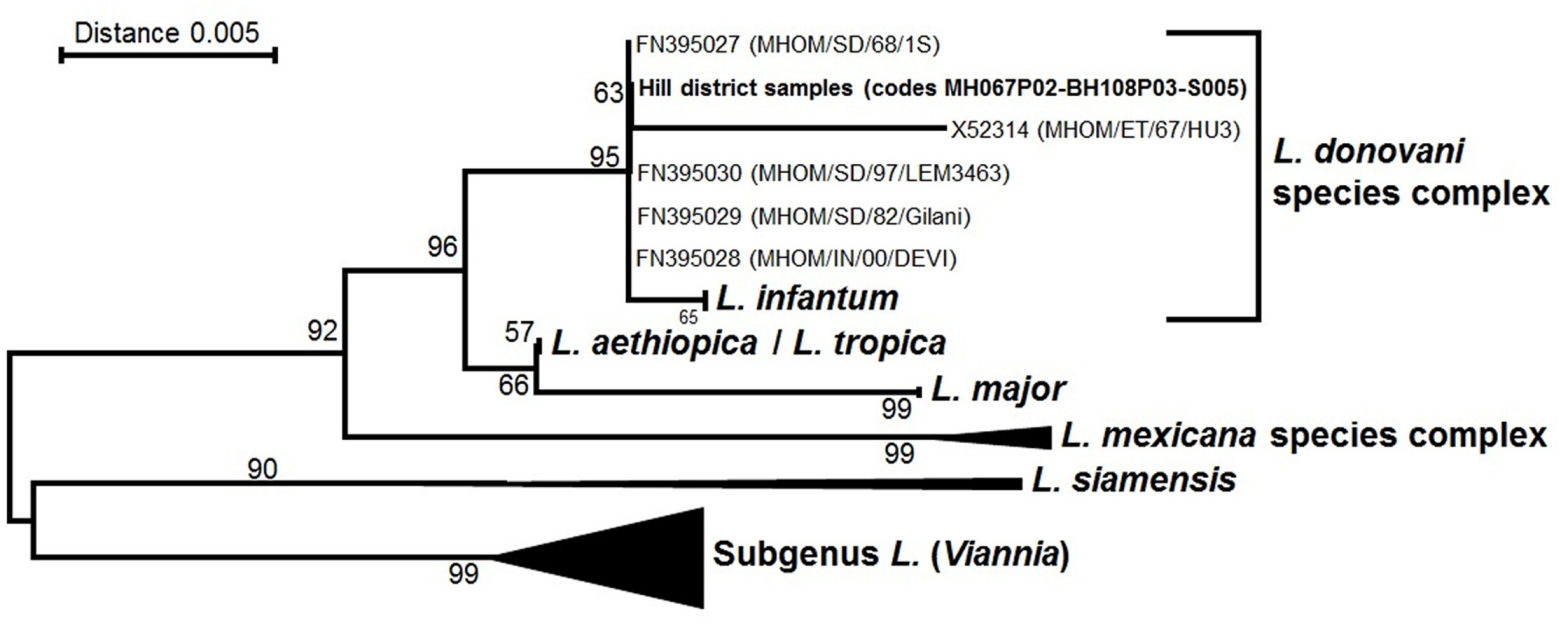
Dendrogram of *hsp70* sequencing.

Fig 3 shows the dendrogram with the location of the three hsp70-positive samples.

## Discussion

Our data show there is strong evidence for local transmission of VL in the hill districts of eastern Nepal. Exposure to known endemic areas in the plains of Nepal or India was not a risk factor for VL. On the contrary, the strongest risk factor for being a VL case in the hills was having a VL case in the close neighborhood of the house (< 100 m). Seventy-five percent of the VL cases (26/35) had no reported exposure to known endemic areas in Nepal or India whatsoever. VL cases actually had travelled less than controls to those areas, though this difference was not statistically significant (adjusted OR. 0.73; 95% C.I. 0.27–1.87; *P* = 0.524).

Entomological and microbiological findings meanwhile strongly point towards local transmission. We caught *P*. *argentipes* sand flies in five of the six villages, at altitudes up to 1500 m above sea level, way above the 700 meter limit suggested in literature [4–6]. In one of the *P*. *argentipes* samples, we were able to demonstrate the presence of *L*. *donovani* DNA. While these entomological data suggest the high likelihood of P. argentipes as the vector of VL in the study areas, it does not unequivocally prove that the species is the vector in this habitat, which would require the demonstration of transmissible parasite stages (e.g. by microscopical observation of *Leishmania* in the sand flies, or detection of stage-specific antigens or RNA) [28].

In the asymptomatic human samples, 24 (5.4%) were positive with SSU-rDNA PCR. This assay was designed for detection of *Leishmania* sp, but can also give positive results with some monoxenous trypanosomatids like *Crithidia* and *Leptomonas* [29]. The latter protozoa are occasionally reported in humans, essentially in cases of immuno-suppression due to HIV or co-infection with *L*. *donovani* [30]

Two of the samples were confirmed as *L*. *donovani*, in three additional ones a *Leishmania sp*. SSU-rDNA signature was demonstrated. Moreover, a *Leishmania* SSU-rDNA sequence was confirmed in 11 out of 16 SSU-rDNA PCR positive sand flies, one of which was shown to be *L*. *donovani*. Among the samples that could not be sequenced, we cannot strictly exclude the occurrence of Leptomonas sp. It should be noted that some trypanosomatids share the *Leishmania* SSU-rDNA sequence, but as far as we know these are not endemic in the Indian subcontinent. In all, these arguments point to active *Leishmania* transmission in the sampled areas.

Finally, the large proportion of the inhabitants showing antibodies to the DAT is strongly supportive for local transmission. DAT has been shown to be negative in healthy populations from Indian states without VL transmission [31]. The DAT prevalence in individuals without a history of VL was 9.6% in our household study, comparable with prevalences in villages with recent outbreaks in the endemic areas of the *Terai* [32]. DAT positivity was present here in all age groups, and not related with travel: among the 139 controls in the case-control study, there were nine asymptomatic DAT positive cases, but only two of them had a history of traveling to VL-endemic territory. Furthermore, there was a clear link between DAT positivity and the presence of VL case(s) in the household, an epidemiological feature of endemic transmission [33]: in our study, having a VL case in the family represented an OR of 3.2 (95% C.I.: 1.643–6.361) (p = 0.0007) to be DAT-positive.

We observed a substantial proportion of domestic animals with positive serological titers and PCR-positivity, as we did previously in the Terai plains of Nepal [34,35]. For the DAT we chose to use one single, relatively high cut-off of 1:1600, regardless of the animal species (e.g. in dogs, 1:800 is the cut-off commonly used), to increase specificity. The DAT seroprevalence (12%) and SSU-rDNA PCR positivity (12%, *Leishmania* sp confirmed by sequencing in 4 samples) amongst goats clearly indicates local exposure to *Leishmania* parasites, as all goats are bred locally. It is tempting to conclude to *L*. *donovani* transmission as the causal agent, as domestic animals are also exposed to *P*. *argentipes* bites, who are opportunistic feeders. Nonetheless, circulation of animal *Leishmania* spp., can be another explanation as titers were low and none of the PCR positives could be confirmed as *L*. *donovani*.

We conclude there is local transmission of *Leishmania donovani* ongoing in the hilly districts of Nepal, based on three arguments. Firstly, there were several VL cases in permanent residents of settlements in the hills, most of them relatively recent, who had not travelled to known endemic areas. Secondly, a considerable number of asymptomatic residents have been exposed to *Leishmania* sp. as shown by DAT serology and PCR and in two of them *L*. *donovani* was confirmed. Thirdly, *P*. *argentipes* sand flies are present in these hilly districts. A substantial number were infected with *Leishmania sp*., and we confirmed *L*. *donovani* at species level in one specimen. Further work is required for a finer genotyping of *L*. *donovani* and to examine links with parasite populations encountered in the lowlands.

The hill districts Okhaldhunga and Bhojpur in Nepal are hitherto not considered as endemic for VL transmission in Nepal, which implies that the health staff is not specifically trained, diagnostic and treatment facilities are not available, reporting is not standardized, and there are no prevention campaigns. Those engaged in VL elimination should pay attention to these new geographical presentations and reconsider the existing risk maps.

## Supporting Information

S1 Fig Study clusters in Okhaldhunga & Bhojpur districts. Okhaldhunga and Bhojpur. Bhojpur and Okhaldhunga are two non-adjacent districts located in the eastern hilly region of Nepal, north of the Terai The range of hills make the transition between the tropical plains and the high mountains of the Himalaya. They shift in altitude and climate from 300 meters elevation in the lower tropical zone, over an upper tropical (300–1,000 m), a subtropical zone (1,000–2,000), a temperate (2,000 to 3,000 m) to the subalpine zone (3000 to 4000m) (Wikipedia). Map sources: OCHA 2008.
Nepal map: http://en.wikipedia.org/wiki/Administrative_divisions_of_Nepal#/media/File:Nepal_districts.png
Okhadhunga district map: http://upload.wikimedia.org/wikipedia/commons/d/da/NepalOkhaldhungaDistrictmap.png
taken from http://commons.wikimedia.org/wiki/File:NepalOkhaldhungaDistrictmap.png
Bhojpur district map: http://upload.wikimedia.org/wikipedia/commons/8/8f/NepalBhojpurDistrictmap.png
taken from http://commons.wikimedia.org/wiki/File:NepalBhojpurDistrictmap.png
(DOCX)

S2 Fig Age at time of VL: Number of cases by age group for the 35 retrospectively identified VL cases. (TIFF)

S1 Table Characteristics of study households *(N = 122)*. (DOCX)

S2 Table DAT results by cluster. (DOCX)

S3 Table DAT results by age and gender. (DOCX)

S1 Checklist STROBE checklist. (DOCX)

## References

[1] Alvar J, Vélez ID, Bern C, Herrero M, Desjeux P, Cano J et al; WHO Leishmaniasis Control Team. Leishmaniasis worldwide and global estimates of its incidence. PLoS One. 2012;7(5):e35671 10.1371/journal.pone.0035671 22693548 PMC3365071

[2] Swaminath CS, Short HE, Anderson LA. Transmission of Indian kala-azar to man by the bites of Phlebotomus argentipes, ann and brun. 1942. Indian J Med Res. 2006 3;123(3):473–7. 16789343

[3] Bhattarai NR, Van der Auwera G, Rijal S, Picado A, Speybroeck N, Khanal B et al Domestic animals and epidemiology of visceral leishmaniasis, Nepal. Emerg Infect Dis. 2010 2;16(2):231–7. 10.3201/eid1602.090623 20113552 PMC2958000

[4] Lysenko AJ. Distribution of Leishmaniasis in the Old World. Bull World Health Organ 1971;44(4):515–520. 5316978 PMC2427824

[5] Park K (2007) Leishmaniasis Park K ed. Park’s Text Book of Preventive and Social Medicine, 6th ed Jabalpur, India: Banarsidas Bhanot, 256–258.

[6] Bhunia GS, Kesari S, Jeyaram A, Kumar V, Das P. Influence of topography on the endemicity of Kala-azar: a study based on remote sensing and geographical information system. Geospat Health. 2010 5;4(2):155–65. 20503185 10.4081/gh.2010.197

[7] Joshi S, Bajracharya BL, Baral MR. Kala-azar (visceral leishmaniasis) from Khotang. Kathmandu Univ Med J (KUMJ). 2006 Apr-Jun;4(2):232–4 18603904

[8] Pandey BD, Pun SB, Kaneko O, Pandey K, Hirayama K. Case report: Expansion of Visceral Leishmaniasis to the Western Hilly part of Nepal. Am J Trop Med Hyg. 2011 1;84(1):107–8. 10.4269/ajtmh.2011.10-0291 21212211 PMC3005498

[9] Naik SR, Rao PN, Datta DV, Mehta SK, Mahajan RC, Mehta S et al Kala-azar in north-western India: a study of 24 patients. Trans R Soc Trop Med Hyg. 1979;73(1):61–5. 442183 10.1016/0035-9203(79)90131-7

[10] Datta U, Rajwanshi A, Rayat CS, Sakhuja V, Sehgal S. Kala-azar in Himachal Pradesh: a new pocket. J Assoc Physicians India 1984;32(12):1072–3. 6526803

[11] Singh S, Biswas A, Wig N, Aggarwal P, Sood R, Wali JP (1999). A new focus of visceral leishmaniasis in sub-Himalayan (Kumaon) region of northern India. J Commun Dis. 1999 6;31(2):73–7. 10810593

[12] Mahajan SK, Machhan P, Kanga A, Thakur S, Sharma A, Prasher BS et al Kala-azar at high altitude. J Commun Dis. 2004 6;36(2):117–20. 16295673

[13] Yangzom T, Cruz I, Bern C, Argaw D, den Boer M, Vélez ID et al Endemic transmission of visceral leishmaniasis in Bhutan. Am J Trop Med Hyg. 2012 12;87(6):1028–37. 10.4269/ajtmh.2012.12-0211 23091191 PMC3516070

[14] Rao JS, Sharma SK, Bhattacharya D, Saxena NB. Sandfly survey in Nainital and Almora districts of Uttaranchal with particular reference to Phlebotomus argentipes, vector of Kala-azar. J Commun Dis. 2001 3;33(1):7–11. 11898464

[15] Ozbel Y, Sanjoba C, Alten B, Asada M, Depaquit J, Matsumoto Y et al Distribution and ecological aspects of sand fly (Diptera: Psychodidae) species in Sri Lanka. J Vector Ecol. 2011 3;36 Suppl 1:S77–86. 10.1111/j.1948-7134.2011.00115.x 21366784

[16] Picado A, Das ML, Kumar V, Dinesh DS, Rijal S, Singh SP et al Phlebotomus argentipes seasonal pattern in India and Nepal. J Med Entomol. 2010 3;47(2):283–6. 20380311 10.1603/me09175

[17] Lewis DJ. 1982 A taxonomical review of the genus Phlebotomus (Diptera: Psychodidae). Bulletin of British Museum Ent. Series 45 (2):121–209

[18] Jacquet D, Boelaert M, Seaman J, Rijal S, Sundar S, Menten J et al Comparative evaluation of freeze-dried and liquid antigens in the direct agglutination test for serodiagnosis of visceral leishmaniasis (ITMA-DAT/VL). Trop Med Int Health. 2006 12;11(12):1777–84. 17176341 10.1111/j.1365-3156.2006.01743.x

[19] Khanal B, Picado A, Bhattarai NR, Van Der Auwera G, Das ML, Ostyn B et al Spatial analysis of Leishmania donovani exposure in humans and domestic animals in a recent kala azar focus in Nepal. Parasitology. 2010 9;137(11):1597–603. 10.1017/S0031182010000521 20459877

[20] Odiwuor S, Muia A, Magiri C, Maes I, Kirigi G, Dujardin JC et al Identification of Leishmania tropica from micro-foci of cutaneous leishmaniasis in the Kenyan Rift Valley. Pathog. Glob. Health. 2012 7;106(3):159–65. 10.1179/2047773212Y.0000000015 23265373 PMC4001575

[21] Montalvo AM, Fraga J, Maes I, Dujardin JC, Van der Auwera G. Three new sensitive and specific heat-shock protein 70 PCRs for global Leishmania species identification. Eur. J. Clin. Microbiol. Infect. Dis. 2012 7;31(7), 1453–1461. 10.1007/s10096-011-1463-z 22083340

[22] Van der Auwera G, Maes I, De Doncker S, Ravel C, Cnops L, Van Esbroeck M, et al Heat-shock protein 70 gene sequencing for Leishmania species typing in European tropical infectious disease clinics. Euro Surveill. 2013 7;18(30):20543 23929181 10.2807/1560-7917.es2013.18.30.20543

[23] Van der Auwera G, Ravel C, Verweij JJ, Bart A, Schönian G, Felger I. Evaluation of four single-locus markers for Leishmania species discrimination by sequencing. J. Clin. Microbiol. 2014 4;52(4):1098–104. 10.1128/JCM.02936-13 24452158 PMC3993476

[24] Versteirt V, Nagy ZT, Roelants P, Denis L, Breman FC, Damiens D et al Identification of Belgian mosquito species (Diptera: Culicidae) by DNA barcoding. Mol Ecol Resour. 2014 8 20. 10.1111/1755-0998.12318 25143182

[25] Ratnasingham S, Hebert PD. bold: The Barcode of Life Data System (http://www.barcodinglife.org). Mol Ecol Notes. 2007 5 1;7(3):355–364. 18784790 10.1111/j.1471-8286.2007.01678.x PMC1890991

[26] R Core Team (2013). R: A language and environment for statistical computing. R Foundation for Statistical Computing, Vienna, Austria http://www.R-project.org/.

[27] Keogh RH and Cox DR. (2014) Case-control studies Institute of Mathematical Statistics Monographs 4; Epidemiologic Methods. Cambridge University Press, UK, Cambridge.

[28] Deborggraeve S, Boelaert M, Rijal S, De Doncker S, Dujardin JC, Herdewijn P et al Diagnostic accuracy of a new Leishmania PCR for clinical visceral leishmaniasis in Nepal and its role in diagnosis of disease. Trop Med Int Health. 2008 11;13(11):1378–83. 10.1111/j.1365-3156.2008.02154.x 18803611

[29] Seblova V, Sadlova J, Carpenter S, Volf P. Speculations on biting midges and other bloodsucking arthropods as alternative vectors of Leishmania. Parasit Vectors. 2014 5 14;7:222 10.1186/1756-3305-7-222 24884857 PMC4024269

[30] Srivastava P, Prajapati VK, Vanaerschot M, Van der Auwera G, Dujardin JC, Sundar S. Detection of Leptomonas sp. parasites in clinical isolates of Kala-azar patients from India. Infect Genet Evol. 2010 10;10(7):1145–50 10.1016/j.meegid.2010.07.009 20633704 PMC2933273

[31] Sundar S, Singh RK, Maurya R, Kumar B, Chhabra A, Singh V et al Serological diagnosis of Indian visceral leishmaniasis: direct agglutination test versus rK39 strip test. Trans R Soc Trop Med Hyg 2006 6;100 (6):533–537. 16325874 10.1016/j.trstmh.2005.08.018

[32] Rijal S, Uranw S, Chappuis F, Picado A, Khanal B, Paudel IS et al Epidemiology of Leishmania donovani infection in high-transmission foci in Nepal. Trop Med Int Health. 2010 7;15 Suppl 2:21–8. 10.1111/j.1365-3156.2010.02518.x 20487421

[33] Bern C, Courtenay O, Alvar J. Of cattle, sand flies and men: a systematic review of risk factor analyses for South Asian visceral leishmaniasis and implications for elimination. PLoS Negl Trop Dis. 2010 2 9;4(2):e599 10.1371/journal.pntd.0000599 20161727 PMC2817719

[34] Bhattarai NR, Van der Auwera G, Khanal B, De Doncker S, Rijal S, Das ML et al PCR and direct agglutination as Leishmania infection markers among healthy Nepalese subjects living in areas endemic for Kala-Azar. Trop Med Int Health. 2009 4;14(4):404–11. 10.1111/j.1365-3156.2009.02242.x 19228350

[35] Khanal B, Rijal S, Ostyn B, Picado A, Gidwani K, Menten J et al Serological markers for leishmania donovani infection in Nepal: Agreement between direct agglutination test and rK39 ELISA. Trop Med Int Health. 2010 11;15(11):1390–4. 21998875 10.1111/j.1365-3156.2010.02631.x

