# Supplementary material for: Transmission of Leishmania donovani in the Hills of Eastern Nepal, an Outbreak Investigation in Okhaldhunga and Bhojpur Districts
Source: PLoS Negl Trop Dis. 2015 Aug 7;9(8):e0003966. doi: 10.1371/journal.pntd.0003966 (PMC4529159; doi:10.1371/journal.pntd.0003966)

# Supporting Information

**S1 Fig. Study clusters in Okhaldhunga & Bhojpur districts.** Okhaldhunga and Bhojpur. Bhojpur and Okhaldhunga are two non-adjacent districts located in the eastern hilly region of Nepal, north of the Terai The range of hills make the transition between the tropical plains and the high mountains of the Himalaya. They shift in altitude and climate from 300 meters elevation in the lower tropical zone, over an upper tropical (300 - 1,000 m), a subtropical zone (1,000 - 2,000), a temperate (2,000 to 3,000 m) to the subalpine zone (3000 to 4000m) (Wikipedia). Map sources:

Nepal map

<http://en.wikipedia.org/wiki/Administrative_divisions_of_Nepal#/media/File:Nepal_districts.png>

Okhadhunga district map

<http://upload.wikimedia.org/wikipedia/commons/d/da/NepalOkhaldhungaDistrictmap.png>

taken from <http://commons.wikimedia.org/wiki/File:NepalOkhaldhungaDistrictmap.png>

Bhojpur district map

<http://upload.wikimedia.org/wikipedia/commons/8/8f/NepalBhojpurDistrictmap.png>

taken from <http://commons.wikimedia.org/wiki/File:NepalBhojpurDistrictmap.png>


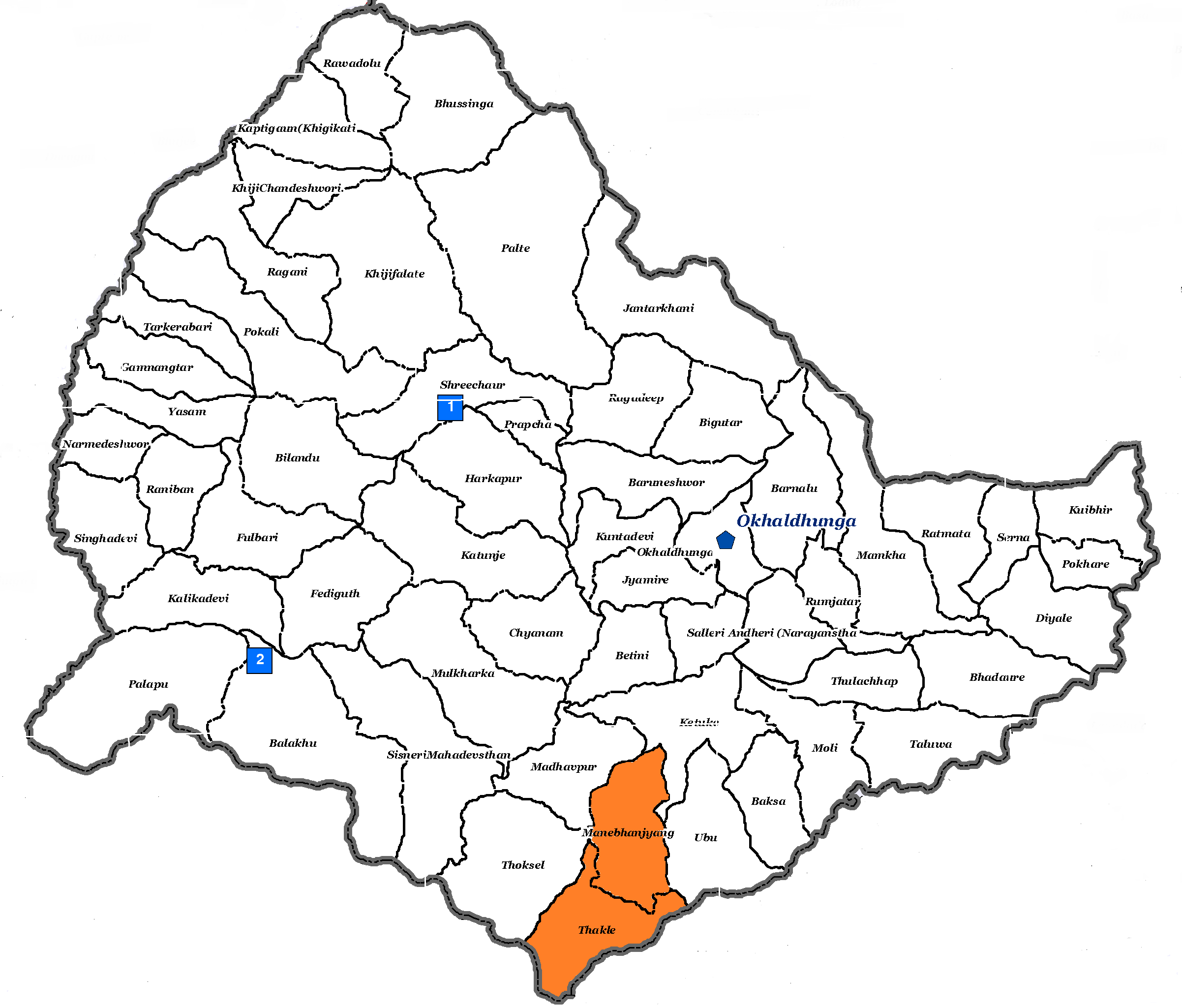

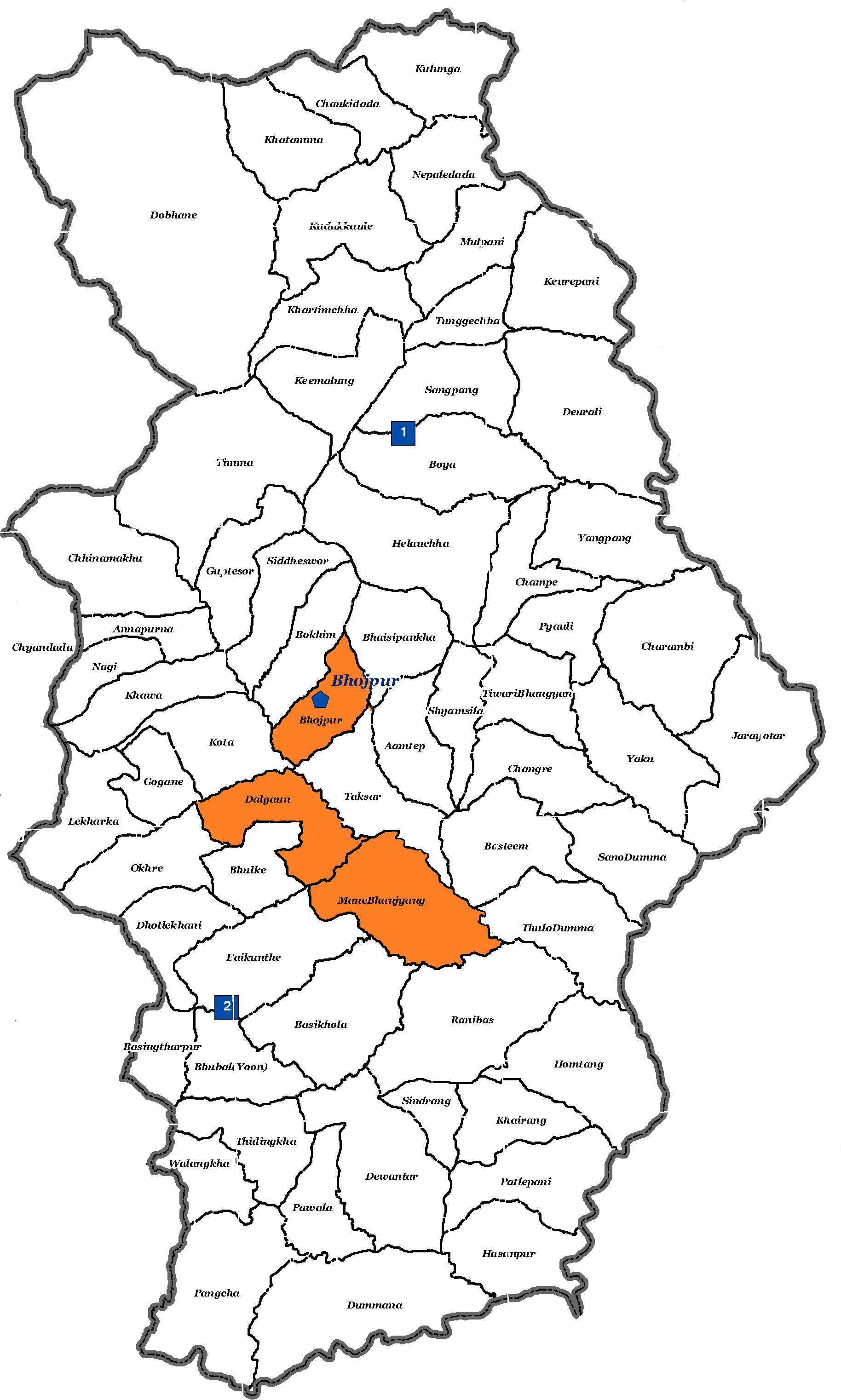

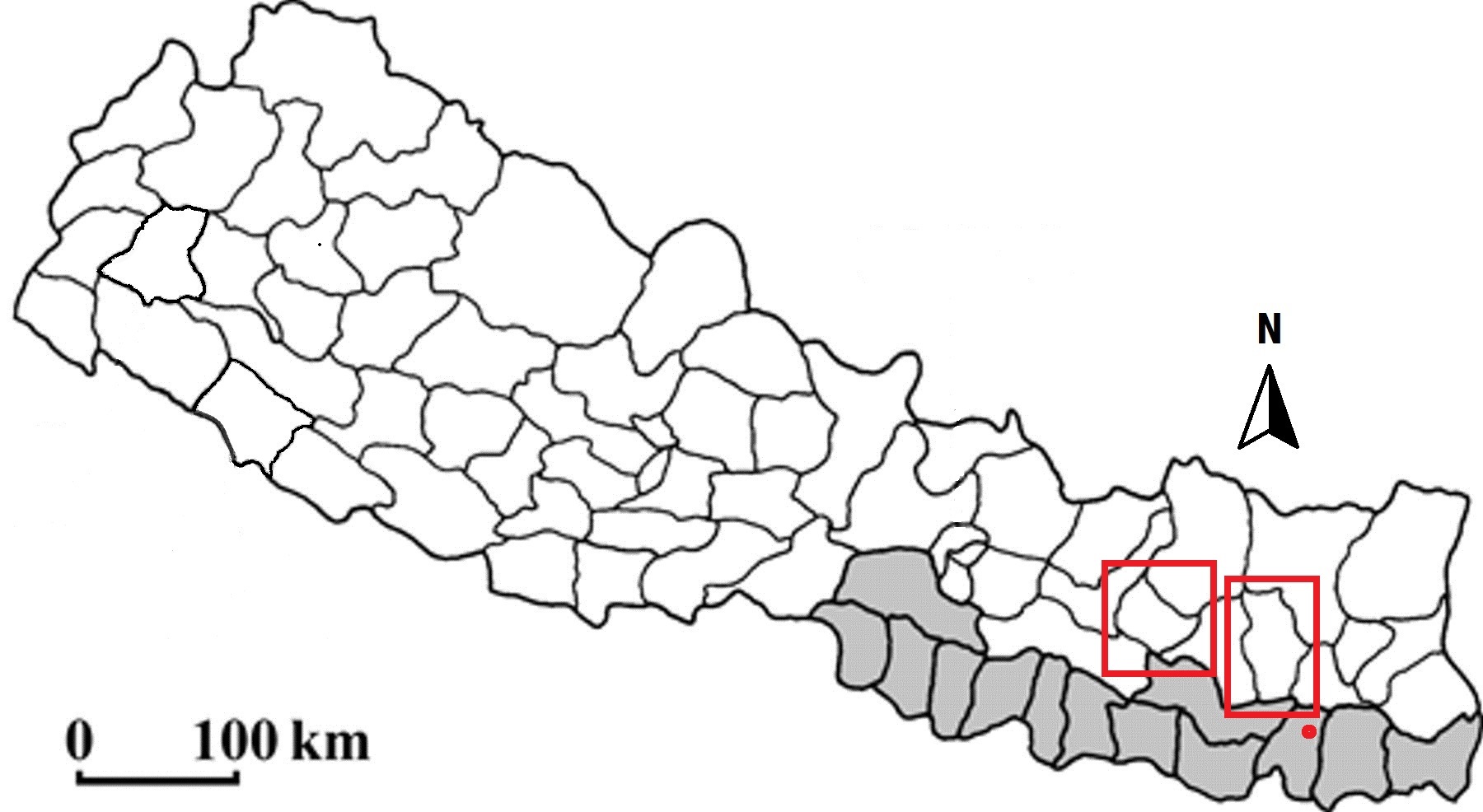


Bhojpur District

Okhaldhunga District

Intended final result:


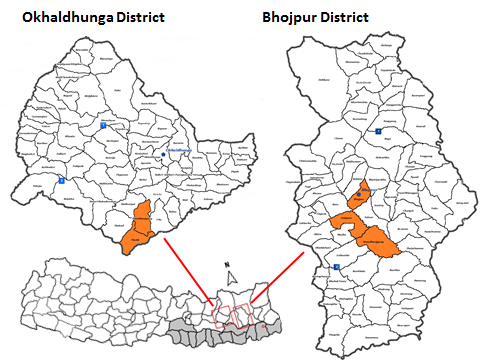

Supplement: S1 Fig — Okhaldhunga and Bhojpur. Bhojpur and Okhaldhunga are two non-adjacent districts located in the eastern hilly region of Nepal, north of the Terai The range of hills make the transition between the tropical plains and the high mountains of the Himalaya. They shift in altitude and climate from 300 meters elevation in the lower tropical zone, over an upper tropical (300–1,000 m), a subtropical zone (1,000–2,000), a temperate (2,000 to 3,000 m) to the subalpine zone (3000 to 4000m) (Wikipedia). Map sources: OCHA 2008. Nepal map: http://en.wikipedia.org/wiki/Administrative_divisions_of_Nepal#/media/File:Nepal_districts.png Okhadhunga district map: http://upload.wikimedia.org/wikipedia/commons/d/da/NepalOkhaldhungaDistrictmap.png taken from http://commons.wikimedia.org/wiki/File:NepalOkhaldhungaDistrictmap.png Bhojpur district map: http://upload.wikimedia.org/wikipedia/commons/8/8f/NepalBhojpurDistrictmap.png taken from http://commons.wikimedia.org/wiki/File:NepalBhojpurDistrictmap.png (DOCX) [file pntd.0003966.s001.docx]
