# Supplementary figures and images for: Transmission of Leishmania donovani in the Hills of Eastern Nepal, an Outbreak Investigation in Okhaldhunga and Bhojpur Districts
Source: PLoS Negl Trop Dis. 2015 Aug 7;9(8):e0003966. doi: 10.1371/journal.pntd.0003966 (PMC4529159; doi:10.1371/journal.pntd.0003966)

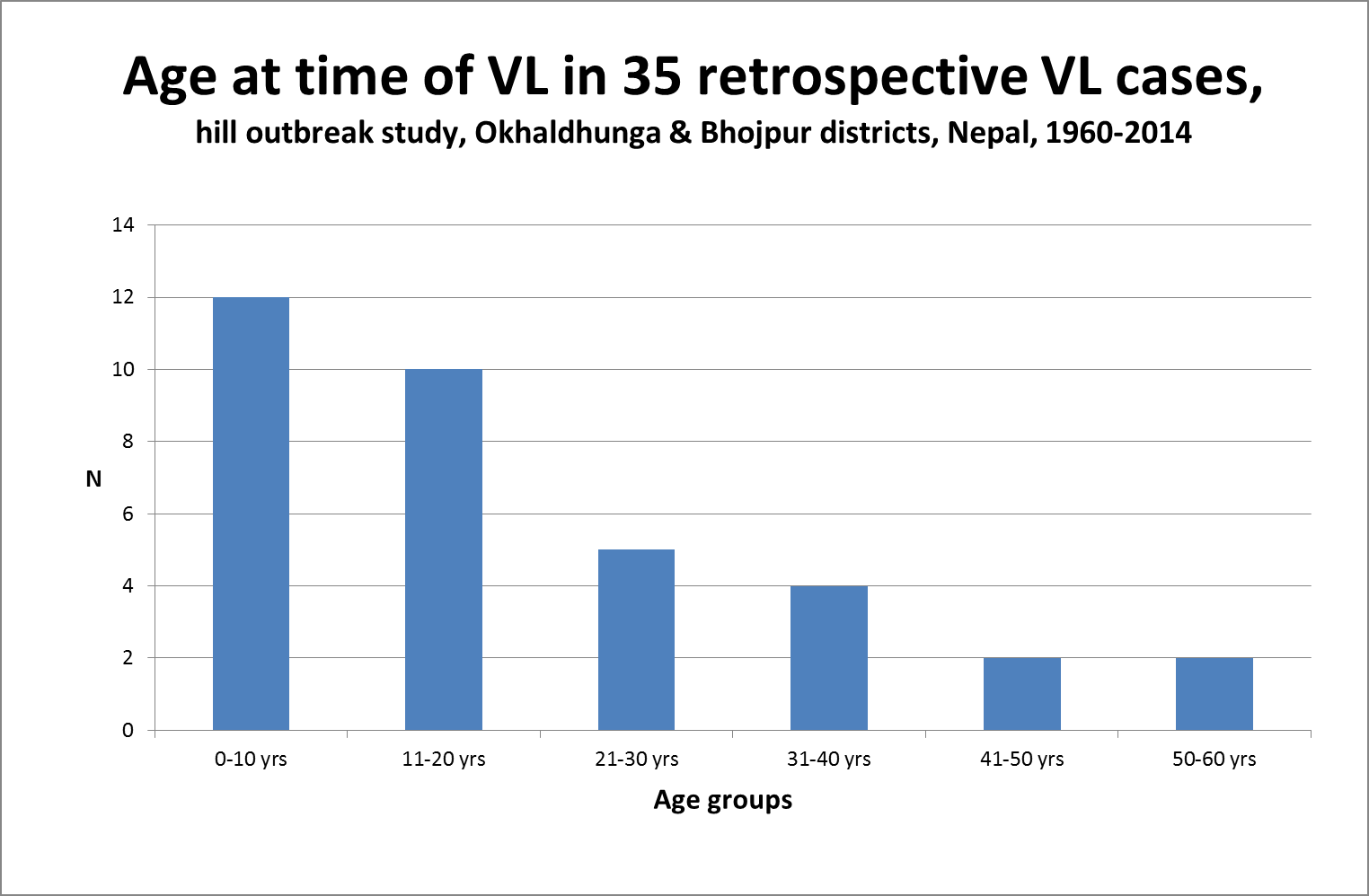

Supplement: S2 Fig — (TIFF) [file pntd.0003966.s002.tiff]
