# Supplementary material for: Transmission of Leishmania donovani in the Hills of Eastern Nepal, an Outbreak Investigation in Okhaldhunga and Bhojpur Districts
Source: PLoS Negl Trop Dis. 2015 Aug 7;9(8):e0003966. doi: 10.1371/journal.pntd.0003966 (PMC4529159; doi:10.1371/journal.pntd.0003966)
Supplement: S1 Table — (DOCX) [file pntd.0003966.s003.docx]

**S1 Table. Characteristics of study households *(N=122)*.**

| **Household Characteristics** | **N (%)** |
| --- | --- |
|  |  |
| **Gender of Head of Household,** N (%) |  |
| Male | 96 (78.7) |
| Female | 26 (21.3) |
|  |  |
| **Age of Head of Household,** Mean (SD) | 48.67 (13.6) |
| Range (years) | (22 - 81) |
|  |  |
| **Religion of** **Head of Household,** N (%) |  |
| Hindu | 84 (68.8) |
| Kirat | 20 (16.4) |
| Christian | 18 (14.8) |
|  |  |
| **Education of** **Head of Household,** N (%) |  |
| Illiterate | 78 (63.32) |
| Primary School | 26 (21.31) |
| More than primary | 18 (15.46) |
|  |  |
| **Occupation of Head of Household,** N(%) |  |
| Farmer | 70 (57.4) |
| Business/ Service/ Skilled worker | 32 (26.2) |
| Unskilled worker | 20 (16.4) |
|  |  |
| **Type of House,** N(%) |  |
| Thatched | 45 (36.88) |
| Mud (Kaccha) | 54 (44.26) |
| Mixed cement with others (Semi Pakka) | 14 (11.48) |
| Wooden | 9 (7.38) |
|  |  |
| **Number of people living in house**, Mean(SD) | 5.67 (2.82) |
| Range | (1 - 13) |
|  |  |
| **Households using bed net**, N (%) | 27 (22.13) |
|  |  |
|  |  |
| **Household with domestic animals, N (%)** | 112 (91.8) |
| Cow, N(%) (median, range) *owners only*  Buffalo, N (%)  Goat, N (%)  Pig, N (%)  Poultry, N (%)  Dog, N (%)  Birds, N (%) | 92 (75.4%) (4, 1-16)  65 (53.3%) (2, 1-7)  102 (83.6%) (8.5, 1-23)  65 (53.3%) (1, 1-12)  95 (77.8%) (6, 1-50)  39 (32.8%) (1, 1-7)  29 (23.8%) (6, 1-20) |
|  |  |
| **Households with past VL cases, N (%)** | 26 (21.31) |
| Households with 1 VL case, N (%) | 20 (16.39) |
| Households with 2 VL cases, N (%)  Households with 3 VL cases, N(%) | 3 (2.46)  3 (2.46) |
