# Supplementary material for: Transmission of Leishmania donovani in the Hills of Eastern Nepal, an Outbreak Investigation in Okhaldhunga and Bhojpur Districts
Source: PLoS Negl Trop Dis. 2015 Aug 7;9(8):e0003966. doi: 10.1371/journal.pntd.0003966 (PMC4529159; doi:10.1371/journal.pntd.0003966)
Supplement: S2 Table — (DOCX) [file pntd.0003966.s004.docx]

**S2 Table. DAT results by cluster.**

| Study clusters | Tested population | VL cases | DAT positives | Non-VL cases | DAT positives (%) |
| --- | --- | --- | --- | --- | --- |
| Jakma | 111 | 3 | 3 (100%) | 108 | 6 (5.6%) |
| Richuwa | 129 | 8 | 8 (100%) | 121 | 16 (13.2%) |
| Mathilo Richuwa | 114 | 7 | 7 (100%) | 107 | 9 (8.4%) |
| Dalgaun WN # 3 | 25 | 0 | 0 | 25 | 1 (4%) |
| Manebhanjyang- 9 | 22 | 3 | 3 (100) | 19 | 8 (42%) |
| Bhojpur WN # 3 | 40 | 2 | 1^a^ | 38 | 0 |
| **TOTAL** | **441** | **23** | **22** | **416** | **40 (9.6%)** |

^a^ A 43-year old man with VL in 1989 was DAT-negative.
