# Supplementary material for: Transmission of Leishmania donovani in the Hills of Eastern Nepal, an Outbreak Investigation in Okhaldhunga and Bhojpur Districts
Source: PLoS Negl Trop Dis. 2015 Aug 7;9(8):e0003966. doi: 10.1371/journal.pntd.0003966 (PMC4529159; doi:10.1371/journal.pntd.0003966)
Supplement: S3 Table — (DOCX) [file pntd.0003966.s005.docx]

**S3 Table. DAT results by age and gender.**

| Age groups | Tested population | VL cases | DAT positives | Non-VL cases | DAT positives (%) |
| --- | --- | --- | --- | --- | --- |
| 2-5 | 31 | 1 | 1 | 30 | 2 (6.7%) |
| 6-15 | 136 | 8 | 8 | 128 | 11 (8.6%) |
| 16-25 | 76 | 4 | 4 | 72 | 7 (9.7%) |
| 26-35 | 56 | 1 | 1 | 55 | 7 (12.7%) |
| 36-45 | 40 | 5 | 4 | 35 | 6 (16.7%) |
| >45 | 102 | 4 | 4 | 98 | 7 (7.2%) |
| **TOTAL** | **441** | **23** | **22** | **418** | **40 (9.6%)** |
| **male** | **193** | **13** | **12** | **180** | **18 (10%)** |
| **female** | **248** | **10** | **10** | **238** | **22 (9.2%)** |
